# Vasopressin-Independent Regulation of Aquaporin-2 by Tamoxifen in Kidney Collecting Ducts

**DOI:** 10.3389/fphys.2019.00948

**Published:** 2019-08-09

**Authors:** Stine Julie Tingskov, Hyo-Jung Choi, Mikkel R. Holst, Shan Hu, Chunling Li, Weidong Wang, Jørgen Frøkiær, Lene N. Nejsum, Tae-Hwan Kwon, Rikke Nørregaard

**Affiliations:** ^1^Department of Clinical Medicine, Aarhus University, Aarhus, Denmark; ^2^Department of Biochemistry and Cell Biology, School of Medicine, Kyungpook National University, Daegu, South Korea; ^3^Zhongshan School of Medicine, Institute of Hypertension, Sun Yat-sen University, Guangzhou, China

**Keywords:** Aquaporin-2, inner medullary collecting duct, tamoxifen, unilateral ureteral obstruction, vasopressin

## Abstract

Arginine vasopressin (AVP) mediates water reabsorption in the kidney collecting ducts through regulation of aquaporin-2 (AQP2). Also, estrogen has been known to regulate AQP2. Consistently, we previously demonstrated that tamoxifen (TAM), a selective estrogen receptor modulator, attenuates the downregulation of AQP2 in lithium-induced nephrogenic diabetes insipidus (NDI). In this study, we investigated the AVP-independent regulation of AQP2 by TAM and the therapeutic effect of TAM on the dysregulation of AQP2 and impaired urinary concentration in a unilateral ureteral obstruction (UUO) model. Primary cultured inner medullary collecting duct (IMCD) cells from kidneys of male Sprague-Dawley rats were treated with TAM. Rats subjected to 7 days of UUO were treated with TAM by oral gavage. Changes of intracellular trafficking and expression of AQP2 were evaluated by quantitative PCR, Western blotting, and immunohistochemistry. TAM induced AQP2 protein expression and intracellular trafficking in primary cultured IMCD cells, which were independent of the vasopressin V2 receptor (V2R) and cAMP activation, the critical pathways involved in AVP-stimulated regulation of AQP2. TAM attenuated the downregulation of AQP2 in TGF-β treated IMCD cells and IMCD suspensions prepared from UUO rats. TAM administration *in vivo* attenuated the downregulation of AQP2, associated with an improvement of urinary concentration in UUO rats. In addition, TAM increased CaMKII expression, suggesting that calmodulin signaling pathway is likely to be involved in the TAM-mediated AQP2 regulation. In conclusion, TAM is involved in AQP2 regulation in a vasopressin-independent manner and improves urinary concentration by attenuating the downregulation of AQP2 and maintaining intracellular trafficking in UUO.

## Introduction

The kidney collecting duct is a critical tubular segment for the arginine vasopressin (AVP)-mediated water reabsorption and regulation of body water homeostasis. The water channel aquaporin-2 (AQP2), playing a central role in the osmotic permeability of the collecting duct to water, is essential for urine concentration (Nielsen et al., 2002; Knepper et al., 2015). Water transport in the collecting duct principal cells is mainly regulated by the antidiuretic hormone, AVP. AVP binds to its receptor, vasopressin type-2 receptor (V2R), localized in the basolateral plasma membrane of collecting duct principal cells. This initiates a cascade leading to an increase in cAMP levels and activation of protein kinase A (PKA)-dependent phosphorylation of AQP2 at the serine 256 residue (pS256-AQP2). This is the main pathway of AQP2 trafficking to the apical plasma membrane of the collecting duct principal cells, which increases the osmotic water permeability (Katsura et al., 1997; Kortenoeven and Fenton, 2014; Jung and Kwon, 2016, 2019). However, several other factors/hormones contributing to AQP2 regulation have also been demonstrated over the recent years, thus increasing complexity (Chou et al., 2000; Fenton et al., 2013; Cheema et al., 2015; Milano et al., 2017; Ando and Uchida, 2018; Tingskov et al., 2018; Ranieri et al., 2019). Although cAMP/PKA signaling is a critical regulatory pathway in the AVP-mediated AQP2 expression and trafficking, it has been discovered that modulation of calmodulin, Wnt5, prostaglandin E2, and cGMP pathways also influences AQP2 regulation and trafficking (Bouley et al., 2005; Hoffert et al., 2005; Olesen et al., 2011; Jung and Kwon, 2016, 2019).

We have previously shown that estrogen can affect renal water reabsorption by regulating AQP2 expression and trafficking in collecting ducts in a female ovariectomized (OVX) model (Cheema et al., 2015). In line with this, we have recently demonstrated that a selective estrogen receptor modulator (SERM), i.e., tamoxifen (TAM), attenuates the downregulation of AQP2 protein expression and improves urinary concentration in rats with lithium-induced nephrogenic diabetes insipidus (NDI) (Tingskov et al., 2018). SERMs are synthetic non-steroidal compounds that can interact with estrogen receptors (ERs) and have the capability of acting as either ER agonist or antagonist, depending on the site of action (Lonard and Smith, 2002). TAM exerts its effects *via* binding to the ERs at the same site as estrogen (Catalano et al., 2014) and is known to have anti-estrogenic effect on the mammary gland as well as estrogenic effects on the cardiovascular and skeletal system (Lonard and Smith, 2002; Sugerman, 2013).

Previous studies have shown that AQP2 was significantly downregulated in the kidneys after bilateral as well as unilateral ureteral obstruction injury (Li et al., 2001, 2003; Norregaard et al., 2005, 2007). In addition, it has been shown in human renal biopsy specimens that a reduction of nephron numbers and the presence of interstitial fibrosis reduce the protein abundance of AQP2, compared with a healthy region of the kidneys (Bedford et al., 2003), indicating a causal relationship between the progression of fibrosis and the expression of renal AQP2.

In this study, we investigated the effect of TAM on AQP2 expression and trafficking in the inner medullary collecting duct (IMCD) cells under normal and disease conditions using a unilateral ureteral obstruction (UUO) model, where urinary concentration is impaired. We hypothesized that TAM increases renal AQP2 expression and improves urinary concentration. The aim of the present study was therefore to investigate the effect of TAM on renal AQP2 expression (intracellular trafficking and protein abundance) in primary cultured IMCD cells as well as in Madin-Darby Canine Kidney (MDCK) cells stably expressing AQP2 and the relevant phospho-mimicking mutant AQP2-S256A. Moreover, the effect of TAM treatment was evaluated on urinary concentration and AQP2 expression in the kidney tissues and IMCD tubule suspensions from UUO rats.

## Materials and Methods

### Primary Culture of Inner Medullary Collecting Duct Cells of Rat Kidney

The animal protocols were approved by the Animal Care and Use Committee of the Kyungpook National University, Korea (KNU 2012-10). Primary cultures enriched in IMCD cells were prepared from pathogen-free male Sprague-Dawley rats (200–250 g, Charles River, Seongnam, Korea) (Choi et al., 2012, 2015). Briefly, rats were anesthetized under enflurane inhalation, and kidneys were rapidly removed. After isolating IMCD cell suspension (Stokes et al., 1987), cells were seeded into 12-well plates. Medium was changed every 48 h and IMCD cells were grown in hypertonic culture medium (640 mOsm/KgH_2_O) supplemented with 10% fetal bovine serum at 37°C in 5% CO_2_, 95% air atmosphere for 3 days, and then in fetal bovine serum-free culture medium for 1 additional day before the experiment at day 5. The culture medium was Dulbecco’s Modified Eagle’s Medium/F12 without phenol red, containing 80 mM urea, 130 mM NaCl, 10 mM HEPES, 2 mM L-glutamine, penicillin/streptomycin 10,000 units/ml, 50 nM hydrocortisone, 5 pM 3,3,5-triiodo-thyronine, 1 nM sodium selenate, 5 mg/L transferrin, and 10% fetal bovine serum (pH 7.4, 640 mOsm/KgH_2_O).

### Inner Medullary Collecting Duct Tubule Suspensions

Fresh inner medullary collecting duct (IMCD) tubules were prepared from rat kidneys, as previously described (Stokes et al., 1987; Chou et al., 2004). Rats were anesthetized under enflurane inhalation. Both inner medullas from the kidneys of one rat were dissected, minced, and digested by incubation with digestion solution DMEM/F12 containing collagenase (20 mg/ml) and hyaluronidase (7 mg/ml) at 37°C for 60 min. After incubation, the IMCD tubules were then centrifuged at 1,000 rpm for 5 min, and the supernatant was discarded. The pellet was resuspended in the modified medium (DMEM/F12, and 100 U/ml penicillin G-streptomycin sulfate, 10% FBS). The samples were then incubated with TAM (50 nM, 100 nM) or a vehicle for 6 h. Upon completion of the incubation, protein was collected in RIPA buffer with proteinase cocktails, and the samples were used for immunoblotting as described previously (Park et al., 2013).

### Tamoxifen Treatment and Imaging of Madin-Darby Canine Kidney Cells Stably Expressing AQP2 or AQP2-S256A

Flp-In T-REx MDCK (FTM) cells stably expressing AQP2-wt and the phospho-mimicking mutant AQP2-S256A (Holst and Nejsum, 2019) were grown at 37°C in 5% CO_2_, 95% air atmosphere in Dulbecco’s Modified Eagle Medium with 1 g/L D-glucose (DMEM, Gibco), 10% fetal bovine serum (FBS, Gibco). Cells were induced with 1 ng/ml doxycycline to express AQP2 or AQP2-S256A for 24 h and with TAM (5 nM) or a vehicle for 6 h prior to fixation. Cells were fixed at room temperature with fixation buffer containing 10 mM MES, 3 mM MgCl_2_, 138 mM KCl, 2 mM EGTA, 0.32 M sucrose (pH 6.1), and 4% PFA for 20 min. Cells were permeabilized for 10 min with 0.1% Triton X-100 in blocking buffer containing 3% BSA in PBS, washed and placed in blocking buffer for 20 min. Cells were then stained for AQP2 with 1:100 mouse anti-AQP2 (sc-51,770) for 1 h, washed and stained with 1:400 rabbit anti-mouse-AlexaFluor 647 (Invitrogen), 8 μM Hoechst, and 25 mM FITC-phalloidin. Z-stack imaging of fixed cells was performed on Nikon Eclipse T*i*-E systems equipped with 100× objectives and Zyla sCMOS cameras, controlled by NIS Elements from Nikon. The fluorescence illumination system was CoolLED-pE-300^white^. Fluorescence filter sets for DAPI, GFP/FITCH, and AlexaFluor 647 were used to detect Hoechst, FITCH, and AlexaFluor 647, respectively. Images were analyzed using ImageJ Fiji software (Schindelin et al., 2012). For image presentation, eight planes were summed.

### Experimental Design and Surgical Procedures

All procedures were in accordance with the Danish National Guidelines for care and handling of experimental animals, and carried out in cooperation with a veterinarian. The animal protocols were approved by the Department of Clinical Medicine, Aarhus University, according to the licenses for the use of experimental animals issued by the Danish Ministry of Justice.

Studies were performed on adult male rats with a starting weight of 180.9 ± 1.42 g. Animals had free access to a standard rodent diet (Altromin, Lage, Germany) and tap water. During the experiments, rats were housed in groups of two to three per cage, with a 12:12-h light-dark cycle, at a temperature of 21 ± 2°C, and a humidity of 55 ± 2%.

The rats were divided into four experimental groups: sham and UUO with vehicle treatment (*n* = 10 for each group) and sham and UUO with TAM treatment (*n* = 12 for each group). TAM (Sigma Chemical Co. T5648) was dissolved in 200 μl of ethanol (EtOH) and mixed with 800 μl of corn oil (Sigma Chemical Co.). TAM was administered by daily oral gavages in doses of 50 mg/kg. The dose was chosen according to previous studies (Kim et al., 2014). The treatment was initiated 5 days prior to the UUO operation and continued to 7 days of postoperative periods. Control groups received vehicle (ethanol in corn oil). At day 5, the rats were anesthetized with sevoflurane (3.5% sevoflurane in O_2_/N_2_O mixture) and placed on a heating pad to maintain body temperature at 37°C during surgery. Through a midline abdominal incision, the left ureter was exposed and occluded with a 3–0 silk ligature. Sham operation was performed using the same method without ligation of ureter. After 7 days, the rats were sacrificed by cervical dislocation and the kidneys were removed and prepared for quantitative PCR (QPCR) or Western blotting.

### Blood and Urine Sampling

Seven days after UUO, rats were anesthetized with sevoflurane. At the time of sacrifice, a blood sample from the aortic bifurcation was taken into heparinized tubes. These blood samples were analyzed to determine osmolality using freezing-point depression (Advanced Osmometer, model 3,900; Advanced Instruments, Norwood, MA, and Osmomat 030-D; Gonotec, Berlin, Germany). Before the kidneys were removed, a urine sample was collected from the proximal part of the ureter above the obstruction and the urine osmolality was measured using freezing-point depression (Advanced Osmometer, as above).

### Immunofluorescence Microscopy of Primary Cultured Inner Medullary Collecting Duct Cells

IMCD cells were grown to confluency on semipermeable filter supports in a transwell chamber (0.4 μm pore size, Transwell® Permeable Supports, catalog no. 3460, Corning) for 4 days. On day 5, IMCD cells were subjected to treatment with vehicle, dDAVP (10^−9^ M, basolateral sides only), and TAM (100 nM, both apical and basolateral sides of the cells) for 15 min and then fixed with 3% paraformaldehyde in PBS, pH 7.4 for 20 min at room temperature. After fixation, cells were washed twice in PBS and permeabilized with 0.3% Triton X-100 in PBS at room temperature for 15 min. Cells were washed and incubated with anti-AQP2 antibody (1,400, AB3274, Millipore) in PBS overnight at 4°C, and nuclei were stained with DAPI (D1306, Molecular Probes). After incubation, cells were washed with PBS and incubated with goat-anti-rabbit IgG Alexa Fluor 488 secondary antibody (A11008, Molecular Probes) for 2 h at room temperature. Cells were washed in a hydrophilic mounting medium containing antifading reagent (P36930, Molecular Probes). AQP2 immunolocalization was observed using a laser scanning confocal microscope (Zeiss LSM 5 EXCITER, Jena, Germany).

### cAMP Measurements

Intracellular cAMP levels were measured in the IMCD cells. IMCD cells were cultured to confluence in a 12-well plate for 4 days and treated on day 5. All measurements were carried out in the presence of 1 mM 3-isobutyl-1-methylxanthine (IBMX; Sigma) to inhibit cyclic nucleotide phosphodiesterases. After 10 min of preincubation with 1 mM IBMX, vehicle, forskolin (a cell-permeable activator of adenylyl cyclase, 10^−6^ M), or TAM (100 nM) was added for 15 min or 6 h in the continued presence of IBMX. The cAMP levels in the cell lysates were determined using a competitive enzyme immunoassay kit (Cayman Chemical, Ann Arbor, Ml), and the results were expressed in picomoles per milliliter of cell lysate.

### Protein Isolation and Semiquantitative Immunoblotting

Renal tissue (inner medulla (IM)) was homogenized in dissecting buffer (0.3 M sucrose, 25 mM imidazole, 1 mM EDTA, pH 7.2) containing the following protease inhibitors: phosphatease inhibitor cocktails 2 and 3 (Sigma-Aldrich, St. Louis, MO) and complete mini protease inhibitor cocktail tablets (serine, cysteine, and metalloprotease inhibitor, Roche, Hvidovre, Denmark). The tissue was homogenized for 240 s at 50 Hz by a TissueLyser LT (Qiagen, Hilden, Germany) and then centrifuged at 1000 × *g* at 4°C for 10 min. Gel samples were prepared from the supernatant in Laemmli sample buffer containing 2% SDS. The total protein concentration of the homogenate was measured using a Pierce BCA protein assay kit (Roche).

Protein samples were run on 12% Criterion TGX Precast Gel (Bio-Rad Laboratories, Copenhagen, Denmark). Proteins were transferred to a nitrocellulose membrane (Hybond ECL, GE Healthcare, Hatfield, UK). Afterward, the blots were blocked with 5% nonfat dry milk in PBS-T (80 mM Na_2_HPO_4_, 20 mM NaH_2_PO_4_, 100 mM NaCl, 0.1 Tween 20, adjusted to pH 7.4). After washing with PBS-T, the blots were incubated with primary antibodies overnight at 4°C. GAPDH was used as a loading control in both inner medulla and cortex (data not shown). The membrane was incubated with horseradish peroxidase (HRP)-conjugated secondary antibodies for 1 h at room temperature (P447 and P448, diluted 1:4000, Dako) and afterward the antigen-antibody complex was visualized using enhanced chemiluminescence system (ECL, Amersham ECL Plus, GE Healthcare). All Western blots were normalized to total protein, as measured using Stain-Free technology (Gürtler et al., 2013).

### Primary Antibodies

For Western blot and immunostaining of tissue sections, we used specific antibodies which had been well characterized before, such as AQP2 (H7661) (Nielsen et al., 2006), pS256-AQP2 (K0407) (Christensen et al., 2000), CaMKII (ab34703, Abcam, Cambridge, United Kingdom), β-actin (Cell Signaling Technology, Danvers, MA), ERα (sc-787, Santa Cruz, Texas, USA), and ERβ (sc-390243, Santa Cruz, Texas, USA). Polyclonal goat anti-rabbit IgG horseradish conjugated secondary antibodies were used [P448] (Dako, Glostrup, Denmark).

### Immunohistochemistry

Kidneys were fixed by retrograde perfusion *via* the abdominal aorta with 4% paraformaldehyde in 0.01 M PBS buffer. The removed kidneys were post-fixed for 2 h and washed in PBS. The fixed kidneys were then dehydrated in graded ethanol and left overnight in xylene. The tissue was embedded in paraffin and then sectioned into 2-μm sections on a rotary microtome (Thermo Scientific, Microm HM 355S).

To evaluate the localization of AQP2 and pS256-AQP2, sections were stained by immunoperoxidase labeling. Endogenous peroxidase was blocked in 35% H_2_O_2_ dissolved in methanol. Afterward, the sections were boiled in TEG buffer (1 mmol/L Tris, 0.5 mmol/L ethylene glycol tetraacetic acid, pH 9.0) for 10 min for antigen retrieval. Nonspecific binding of immunoglobulin was prevented by incubating the sections in 50 mmol/L NH_4_Cl for 30 min followed by blocking in PBS supplemented with 1% bovine serum albumin (BSA), 0.2% gelatin, and 0.05% saponin. Sections were incubated with primary antibodies diluted in 0.1% BSA and 0.3% Triton X-100 at 4°C overnight in a humidity chamber. After being rinsed in PBS supplemented with 0.1% BSA, 0.2% gelatin, and 0.05% saponin for 3 min × 10 min, the sections for immunoperoxidase labeling were incubated for 1 h at room temperature with horseradish peroxidase-conjugated secondary antibody. After rinsing, the sections were incubated in 3,3′-diaminobenzidine for 10 min in order to visualize the peroxidase, and counterstained in Mayer’s hematoxylin. Conventional light microscopy was performed using an Olympus BX50 microscope.

### Quantitative PCR

Total RNA was isolated from the kidney cortex with a Nucleospin RNA II mini kit following the manufacturer’s protocol (Macherey Nagel, Düren, Germany). RNA concentration was quantified by spectrophotometry at 260 nm and then stored at −80°C. cDNA synthesis was performed on 0.5 μg of RNA with the AffinityScrips QPCR cDNA synthesis kit (Life Technologies, Thermo Fisher Scientific, Cambridge, MA). For QPCR, 100 ng of cDNA served as the template for PCR amplication using the SYBR® Green QPCR Master Mix according to the manufacturer’s instructions (Life Technologies) running on an Aria Mx3000P qPCR System (Agilent Technologies, Santa Clara, CA). GAPDH was used as control gene. The sequences of the primers used are shown in Table 1.

**Table 1:** Sequences of the primers used for QPCR.

Primer	Sequence
AQP2	Sense 5′-CCC TCT CCA TTG GTT TCT CTG TT-3′ Antisence 5′-TGG ATT CAT GGA GCA ACC G-3′
ER-α	Sense 5′-TAC GAA GTG GGC ATG ATG A-3′ Antisence 5′-AAG GTT GGC AGC TCT CAT GT-3′
ER-β	Sense 5′-TAT CTC CTC CCA GCA GCA GT-3′ Antisence 5′-CTC CAG CAG CAG GTC ATA CA-3′
GPER	Sense 5′-CCC TTG ACA GGC CAC ATA GT-3′ Antisence 5′-CTC CGT GCT GTC TGG TAT GA-3′
GAPDH	Sense 5′-TAA AGG GCA TCC TGG GCT ACA CT-3′ Antisence 5′-TTA CTC CTT GGA GGC CAT GTA GG-3′

### Statistical Analysis

Values are presented as means ± SEM. Multiple comparisons between the *in vivo* experimental groups were performed using Kruskal-Wallis test follow by Dunn’s multiple comparisons test or two-way ANOVA followed by *post hoc* analysis by a Tukey’s multiple comparisons test. Additionally, the IMCD cells were compared using unpaired *t* test. GraphPad Prism software (GraphPad Software, La Jolla, CA) was used for all statistical analysis. *p* < 0.05 was considered significant.

## Results

### Tamoxifen Regulates AQP2 Expression in a Vasopressin-Independent Manner

**Figure 1:**
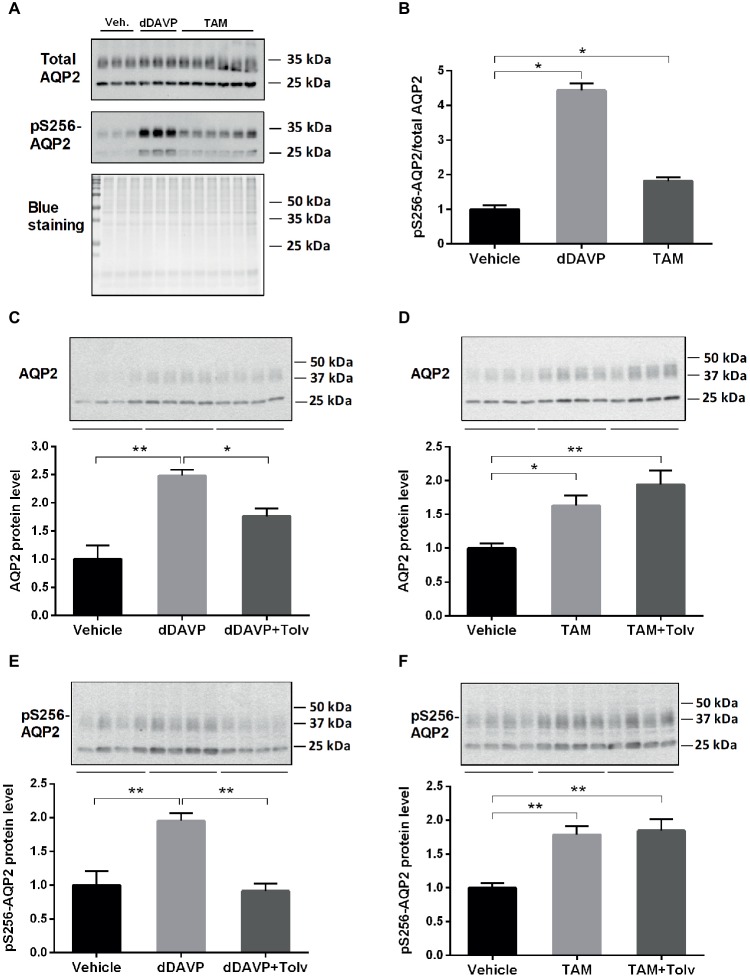
dDAVP, tamoxifen, and tolvaptan affect AQP2 in IMCD cells. **(A,B)** Representative Western blot (WB) analysis of total AQP2 and pS256-AQP2 and ratio between them after treatment with dDAVP (10^−9^ M for 15 min) and TAM (100 nM for 15 min) (*n* = 3–6). **(C,E)** Representative WB analysis of AQP2 and pS256-AQP2 in primary cultured IMCD cells treated with dDAVP (10^−9^ M for 6 h) and tolvaptan (V2R antagonist: 50 nM pre-treatment for 3 h + co-treatment for 6 h) (*n* = 8). **(D,F)** Representative WB analysis of AQP2 and pS256-AQP2 in the primary cultured IMCD cells after treatment with tamoxifen (TAM) (100 nM for 6 h) and tolvaptan (50 nM pre-treatment for 3 h + co-treatment for 6 h) (*n* = 16). Each bar represents mean ± SEM. ^*^p < 0.05 was considered statistically significant. ^**^p < 0.01.

To investigate whether TAM has an effect on AQP2 expression in the collecting duct, we utilized primary cultured IMCD cells from rat kidney. We determined the effect of dDAVP and TAM treatment on expression of total AQP2 and AQP2 phosphorylated at serine 256 (pS256-AQP2) in the primary IMCD cells using semiquantitative immunoblotting (Figure 1). Then we investigated whether TAM-induced changes of AQP2 expression are mediated through an activation of the V2R. IMCD cells were treated with vehicle, dDAVP, or TAM for 15 min (Figures 1A,B) or 6 h (Figures 1C–F). The results revealed that pS256-AQP2 expression (pS256-AQP2/total AQP2) was significantly induced after short-term dDAVP treatment and importantly, short-term TAM treatment also significantly induced pS256-AQP2 expression (pS256-AQP2/total AQP2, Figures 1A,B). Moreover, dDAVP treatment for 6 h induced AQP2 and pS256-AQP2 expression, which was inhibited by co-treatment with V2R antagonist tolvaptan (Figures 1C,E). TAM treatment for 6 h also induced a significant increase of AQP2 and pS256-AQP2 expression; however, this was not inhibited by tolvaptan co-treatment (Figures 1D,F), indicating that the effect of TAM on AQP2 expression is unlikely to be mediated through V2R, but by a vasopressin-independent manner.

### Tamoxifen Induces AQP2 Trafficking to the Plasma Membrane in Primary Cultured Inner Medullary Collecting Duct Cells and Madin-Darby Canine Kidney Cells

**Figure 2:**
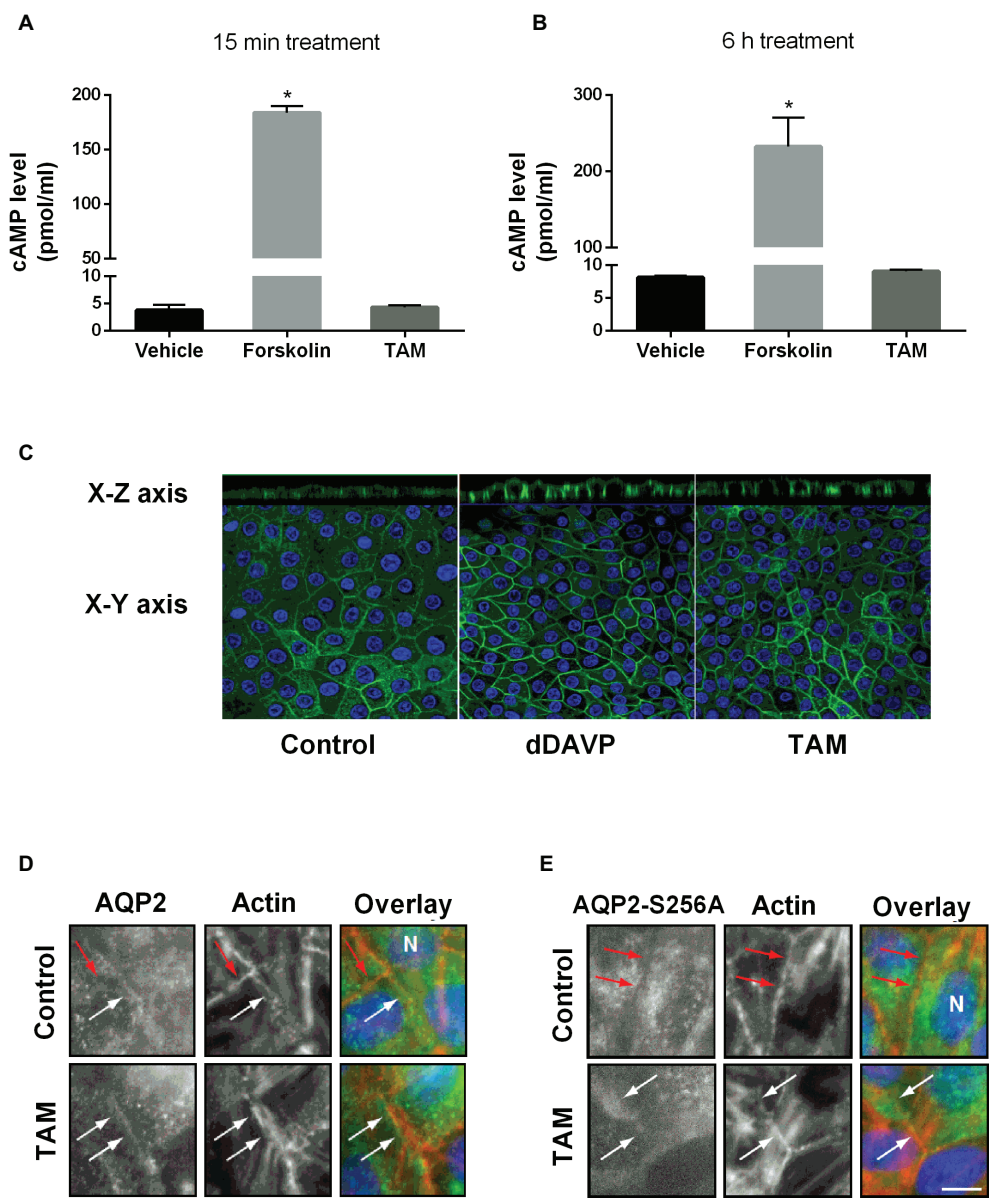
The effect of tamoxifen treatment on intracellular cAMP levels and AQP2 trafficking in IMCD cells and MDCK cells. **(A,B)** Represent intracellular cAMP levels analyzed by ELISA in primary cultured IMCD cells treated with forskolin (10^−6^ M) or tamoxifen (100 nM) for 15 min **(A)** or 6 h **(B)** (*n* = 4). Each bar represents mean ± SEM. ^*^p < 0.05 was considered statistically significant. **(C)** Immunofluorescence staining of AQP2 in primary cultured IMCD cells after treatment with dDAVP (10^−9^ M) for 30 min on the basolateral side or tamoxifen (100 nM) for 30 min on the basolateral side. Green fluorescence represents AQP2 and blue color represents the nuclei. **(D,E)** Representative immunofluorescence staining of AQP2 and AQP2-S256A in MDCK cells stably expressing AQP2 and AQP2-S256A and treated with TAM for 6 h in a concentration of 5 nM, as indicated. Eight planes from a Z-stack were summed for visualization. White arrows indicate AQP2 in the plasma membrane (PM) and red arrows indicate no AQP2 in PM. Scale bar is 10 μm.

Since cAMP/PKA signaling is a major pathway in the AVP-mediated trafficking of AQP2 to the apical membrane, cAMP production was measured in primary cultured IMCD cells after forskolin (10^−6^ M) or TAM (10^−7^ M) treatment for 15 min (Figure 2A) or 6 h (Figure 2B). Forskolin treatment (a cell-permeable activator of adenylyl cyclase) significantly increased intracellular cAMP levels at 15 min and 6 h, whereas TAM had no effect on the production of cAMP (Figures 2A,B), demonstrating that the effect of TAM was not mediated by cAMP/PKA signaling. Next, we examined membrane targeting of AQP2 in primary cultured IMCD cells after treatment with dDAVP (10^−9^ M) or TAM (10^−7^ M) for 15 min. Laser scanning confocal microscopy revealed a distinct membrane labeling of AQP2 after dDAVP or TAM treatment (Figure 2C). In particular, the apical targeting of AQP2 was prominent after dDAVP or TAM treatment, compared with vehicle-treated control, as observed in the x-z images (Figure 2C).

Afterward, we investigated the requirement of phosphorylation of Ser256 for TAM-induced membrane accumulation. As previously reported, the serine (S) 256 mutation to alanine (A), AQP2-S256A mimics constitutively non-phosphorylated AQP2 in S256. AQP2-wt localized to the plasma membrane as well as to the cytoplasm (Figure 2D), whereas AQP2-S256A localized mainly to intracellular structures (Figure 2E) as previously described (Holst and Nejsum, 2019). In the presence of TAM, AQP2-S256A accumulated in the plasma membrane, as did wild-type AQP2 (Figures 2D,E). Thus, TAM-mediated plasma membrane accumulation of AQP2 can occur without phosphorylation of S256.

### Tamoxifen Prevents AQP2 Downregulation in Inner Medullary Collecting Duct Cells Treated With TGF-β and Inner Medullary Collecting Duct Tubule Suspensions From 7dUUO Rats

**Figure 3:**
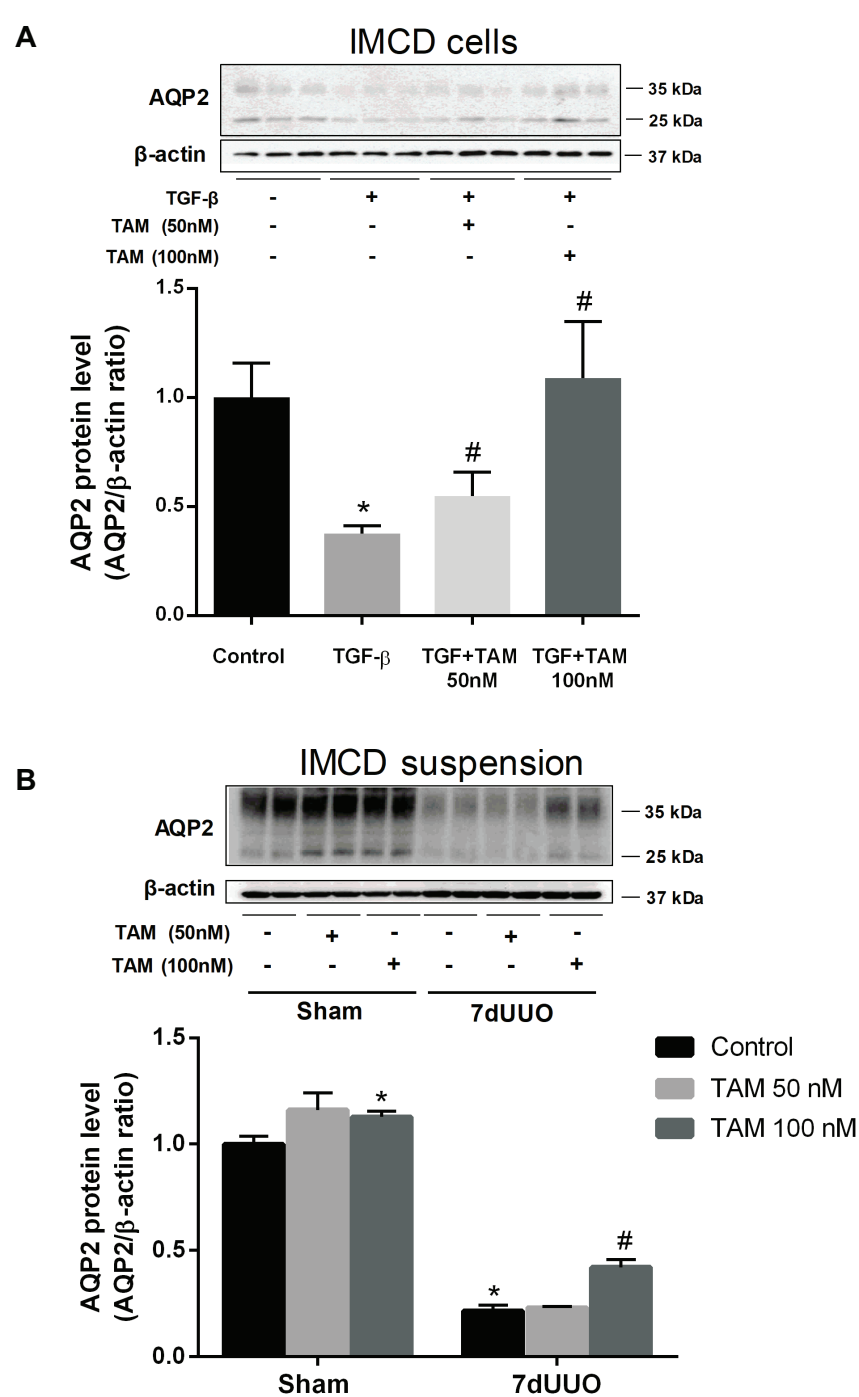
Effects of tamoxifen on AQP2 expression in TGF-β stimulated IMCD cells and IMCD cell suspension from rats subjected to 7dUUO. **(A)** Representative Western blot (WB) of AQP2 and β-actin in IMCD cells stimulated with TGF-β (10 ng/ml) for 48 h and co-treatment with TAM in two different concentrations (50 and 100 nM) for 6 h. **(B)** WB performed for AQP2 in IMCD cell suspension from rats subjected to 7 days of UUO and treated with TAM in two concentrations, respectively, 50 and 100 nM for 6 h and analysis of protein band intensity for AQP2 relative to β-actin. Each bar represents the mean ± SEM (*n* = 4). ^*^p < 0.05 compared to control. ^#^*p* < 0.05 compared to TGF-β or 7dUUO.

To investigate the direct effect of TAM on AQP2 expression in IMCDs which were treated with TGF-β to mimic fibrosis, AQP2 protein levels were measured using Western blotting of primary cultured IMCD cells. We first determined the optimal dose and time of exposure of TGF-β in order to make the IMCD cells fibrotic (data not showed). The most optimal effect was obtained using a concentration of 10 ng/ml of TGF-β for 48 h; therefore, we chose this concentration and time for further experiments. To evaluate the effect of TAM on the primary cultured IMCD cells after treatment with TGF-β, we performed Western blot analysis of FN, α-SMA (data not shown) as well as AQP2. Interestingly, AQP2 expression was markedly reduced in IMCD cells exposed to TGF-β, whereas TAM co-treatment for the last 6 h at a concentration of 100 nM normalized AQP2 protein expression to control level (Figure 3A).

Next, we performed studies of the IMCD tubule suspension obtained from rats subjected to 7dUUO and incubated with TAM in two different concentrations 50 and 100 nM for 6 h (Figure 3B). Western blot analysis showed increased protein levels of AQP2 after treatment with 100 nM TAM compared to the controls. IMCD suspensions prepared from the UUO subjected rats showed significantly reduced expression of AQP2 compared to sham rats. This downregulation was attenuated by TAM treatment (100 nM, 6 h).

### Tamoxifen Attenuates Downregulation of AQP2 in Rats Subjected to Unilateral Ureteral Obstruction

**Figure 4:**
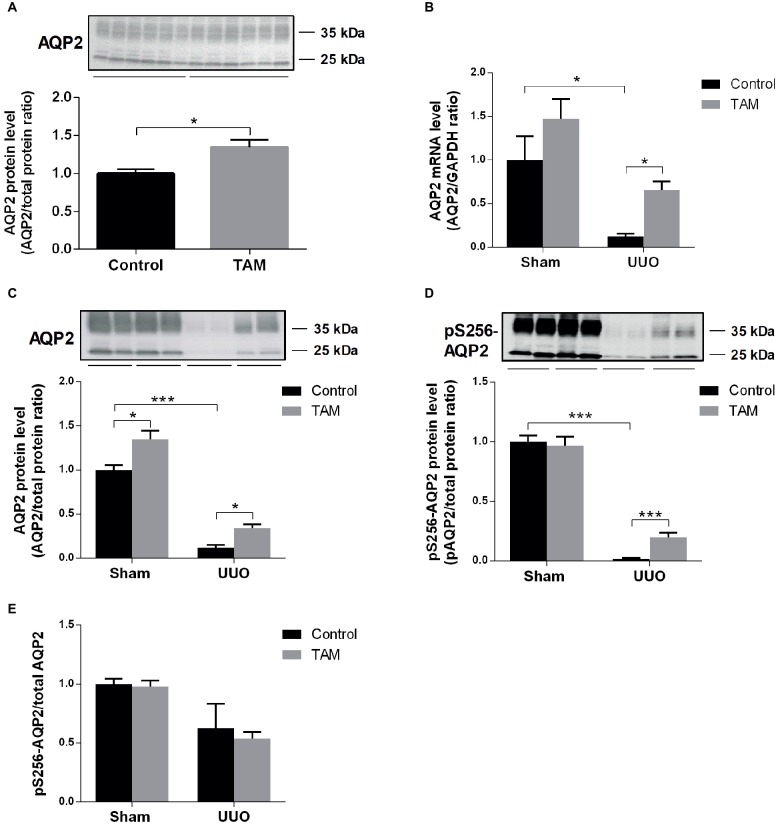
Tamoxifen attenuates downregulation of AQP2 in rat subjected to UUO. **(A)** Representative protein expression of AQP2 in sham-operated rats treated with vehicle and tamoxifen respectively. **(B)** Shows mRNA level of AQP2 relative to GAPDH. **(C,D)** Western blotting performed for protein level of AQP2 and pS256-AQP2 in kidney inner medulla. **(E)** Shows the ratio between pS256-AQP2 and total AQP2. Analysis of protein band intensity relative to total protein (Gürtler et al., 2013). Each bar represents the mean ± SEM (*n* = 6). ^*^p < 0.05was considered statistically significant and ^***^p < 0.001.

Next, we examined the effect of TAM on the expression of inner medullary AQP2 in rats subjected to UUO. First, we were studying the effect of TAM at AQP2 in a normal sham-operated control condition *in vivo*. The protein level of AQP2 was significantly increased in TAM-treated control rats compared to non-treated rats (Figure 4A). Then, we investigated AQP2 at the transcriptional level, which was done by measuring AQP2 mRNA expression. Our data showed reduced mRNA expression of AQP2 in rats subjected to UUO compared to sham rats. Treatment with TAM attenuated the downregulation of AQP2 mRNA expression in the UUO rats (Figure 4B).

Using Western blot analysis, both total AQP2 and pS256-AQP2 protein levels were evaluated in UUO rats. Consistent with the mRNA data, we observed that TAM likewise attenuated the downregulation of AQP2 at the protein level in UUO rats and increased the expression in sham rats (Figure 4C). Furthermore, administration of TAM also decreased the downregulation of the phosphorylated form of AQP2 at S256 in rats subjected to UUO (Figure 4D). Analysis of the ratio of pS256-AQP2 vs. total AQP2 (Figure 4E) demonstrated that TAM did not increase the activation level, but increased the amount of AQP2 available to be phosphorylated.

To investigate the effects of TAM on renal function, urine osmolality was measured in urine collected directly from the proximal ureter just above the obstructed site. The osmolality was significantly higher in the TAM-treated UUO rats compared to vehicle-treated UUO rats (Table 2). There was no change in plasma osmolality among the different groups. Additionally, the kidney weight was significantly higher in obstructed kidneys compared to sham kidneys; however, administration of TAM did not affect kidney weight (Table 2). These data suggest that TAM treatment results in a partial recovery of urinary concentration in the UUO model.

**Table 2:** The effect of tamoxifen on kidney weight, plasma and urine osmolality in UUO rats.

	Sham	Sham TAM	7dUUO	7dUUO TAM
Kidney weight (KW g/100 g BW)	0.35 ± 0.01	0.45 ± 0.01	0.76 ± 0.05^*^	0.76 ± 0.03^#^
Plasma osmolality (mOsmol/kg)	298.8 ± 2.2	296.2 ± 1.5	298.4 ± 2.2	301.9 ± 3.0
Urine osmolality (ureter) (mOsmol/kg)	–	–	310.4 ± 2.7	356.4 ± 6.0^¤^

*Values are presented as mean ± SEM. Sham: n = 6, sham + TAM: n = 6, 7dUUO: n = 9, 7dUUO + TAM: n = 10*.

* *p < 0.05 compared to sham*.

# *p < 0.05 compared to sham + TAM*.

¤ *p < 0.05 compared to 7dUUO*.

### Tamoxifen Affects Membrane Labeling of AQP2 and pS256-AQP2 in Unilateral Ureteral Obstruction Rats

**Figure 5:**
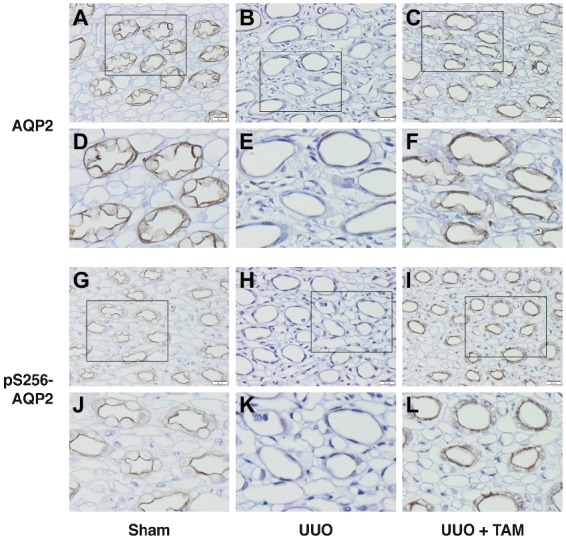
Tamoxifen attenuates reduced labeling of AQP2 in rat subjected to UUO. **(A–F)** Show immunohistochemistry labeling of AQP2 in inner medulla from rats subjected to UUO or sham. **(D–F)** represent the section in the box from **(A–C)** respectively. **(G–L)** Represent pS256-AQP2 in the apical membrane of the inner medullary collecting duct principal cells from sham and UUO subjected rats. **(J–L)** Show the section in the box from **(G–I)** respectively. Magnification: ×40, scale bar: 20 μm.

After analyzing the protein level using Western blotting, we investigated the localization of AQP2 and pS256-AQP2 using immunohistochemical analyses. Immunoperoxidase microscopy demonstrated weak labeling of AQP2 and pS256-AQP2 in the apical membrane of the collecting duct cells in the UUO rats compared to the sham rats (Figures 5A,B,D,E,G,H,J,K). In the UUO rats treated with TAM, however, lesser reduction of the labeling intensity of AQP2 and pS256-AQP2 was seen, compared with vehicle-treated UUO rats (Figures 5C,F,I,L vs. Figures 5B,E,H,K). These results suggested that TAM attenuated the downregulation of AQP2 at both the mRNA and protein level under pathophysiological circumstances, using a UUO model.

### The Effect of Tamoxifen on CaMKII Expression in Unilateral Ureteral Obstruction Rats

**Figure 6:**
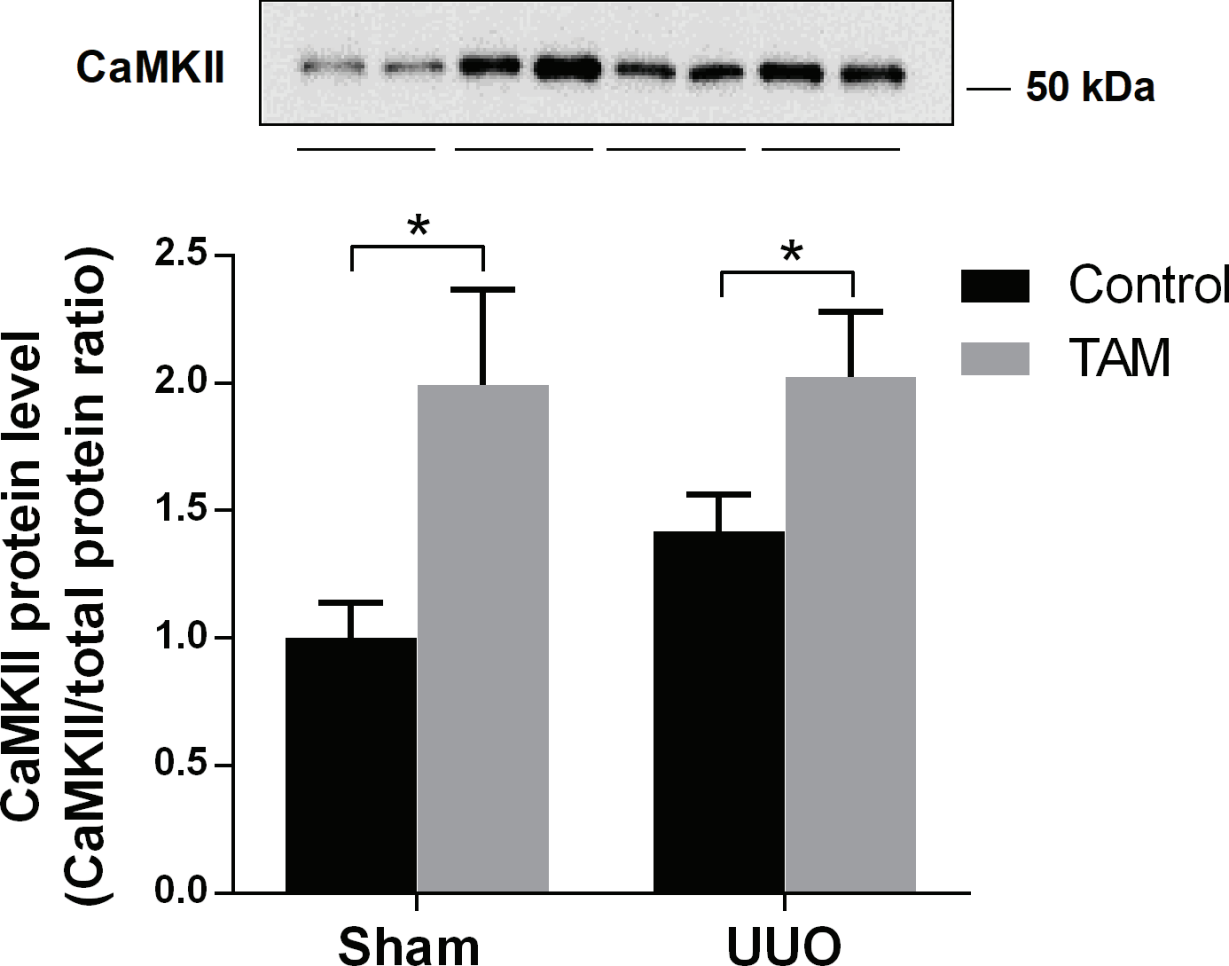
Effects of tamoxifen on calmodulin-dependent protein kinase II (CaMKII) in UUO rats. Represents protein expression of CaMKII in inner medulla from rats subjected to 7dUUO. Each bar represents the mean ± SEM (*n* = 6). ^*^p < 0.05 was considered statistically significant.

It is known that calmodulin-dependent protein kinase II (CaMKII) is capable of phosphorylating AQP2 at S256 (Bradford et al., 2014). In order to investigate a possible pathway for the regulation of AQP2 after TAM treatment, we measured the expression of CaMKII in the inner medulla of the kidneys from rats subjected to sham or UUO. Figure 6 shows that the protein level of CaMKII was significantly increased in TAM-treated sham and UUO rats, suggesting that CaMKII is likely to be involved in TAM-regulated AQP2 expression.

### The Effect of Tamoxifen on the Expression of Estrogen Receptors

**Figure 7:**
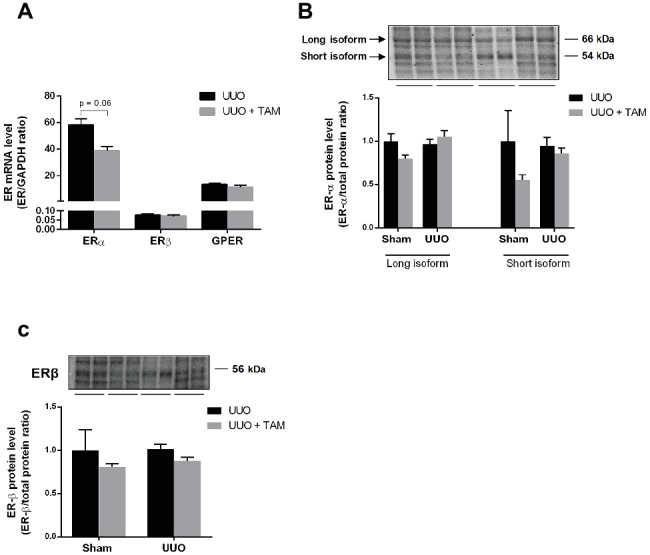
Expression of estrogen receptors in renal tissue from UUO rats. **(A)** Regulation of mRNA expression of ERα, ERβ, and GPER in response to 7dUUO and tamoxifen treatment normalized to GAPDH. **(B)** Represents protein expression of ERα in response to UUO and TAM. The antibody recognizes both the long (66 kDa) and short (54 kDa) isoform of ERα. **(C)** Shows Western blot of ERβ in inner medulla from rats subjected to UUO or sham. Analysis of protein band intensity relative to total protein (Gürtler et al., 2013). Each bar represents the mean ± SEM (*n* = 6).

Since TAM binds to estrogen receptors (ERs), we determined the mRNA expression of ERα, ERβ and the membrane-bound GPER in rats subjected to UUO (Figure 7A). All three ERs were expressed in UUO kidneys and TAM did not affect the expression significantly, although TAM tended to reduce ERα expression. Moreover, protein levels of ERα and ERβ were evaluated using immunoblotting. Data demonstrated that there was no significant difference in the expression of ERα and ERβ after TAM treatment and 7dUUO, although ERα short isoform tended to be reduced by TAM (Figures 7B,C). It has been suggested that ERα contributes to the most estrogen signaling in the kidney (Jelinsky et al., 2003; Irsik et al., 2013) and, as expected, our results showed that ERα is the most prominent ER in the kidney, whereas ERβ is less abundant in the kidney, as previously demonstrated (Wells et al., 2005).

## Discussion

We have recently shown that TAM attenuates the downregulation of AQP2 protein expression and improves urinary concentration in rats with lithium-induced NDI (Tingskov et al., 2018). In this study, we investigated the effect of TAM on the expression and trafficking of AQP2 under normal physiological conditions as well as under pathophysiological circumstances, using a UUO model. The study showed that TAM induces plasma membrane targeting as well as protein expression of AQP2 in collecting duct cells, which contributes to the increased water reabsorption *in vivo*. This effect was not mediated *via* stimulation of the V2R and independent of cAMP-PKA activation; however, phosphorylation of AQP2 at serine 256 was induced. The findings suggest that TAM modulates the membrane targeting and protein expression of AQP2 through bypassing the conventional V2R-cAMP-PKA signaling pathway.

Previous studies have demonstrated that calmodulin plays an important role in AQP2 activation in isolated IMCD and mouse cortical collecting duct mpkCCD cells (Hoffert et al., 2005; Ando et al., 2016). Calmodulin is a calcium-binding protein that regulates numerous target molecules, including calmodulin-dependent protein kinases (CaMK) and phosphatases (Ando and Uchida, 2018). Interestingly, Bayes’ theorem, based on previous transcriptomic and proteomic profiling results, kinase substrate data, and evidence of vasopressin-mediated kinases, identified that CaMKII, PKA, and PKB (Akt) are the candidate protein kinases which phosphorylate AQP2 at serine 256 (Bradford et al., 2014). Our data showed that TAM markedly increases CaMKII expression in inner medullary tissue in both sham-operated control rats as well as in UUO rats, suggesting that the TAM-induced trafficking of AQP2 could possibly be mediated by the CaMKII signaling pathway. Interestingly, our studies also demonstrated that TAM treatment was associated with AQP2-S256A accumulation in the plasma membrane, similar to wild-type AQP2, indicating that TAM-mediated plasma membrane accumulation of AQP2 can occur, despite the absence of phosphorylation of S256. Additionally, previous studies have demonstrated that estrogen activates CaMKII in the mice hippocampus time-dependently (Sawai et al., 2002). Moreover, they proposed that estrogen could have a non-nuclear estrogen-signaling pathway involving a possible direct interaction of ERs with CaMKII (Sawai et al., 2002; Soeda et al., 2011).

Tamoxifen exerts its effect *via* binding to the ERs at the same site as estrogen (Catalano et al., 2014) and has the capability of acting as either agonist or antagonist on the ER, depending on the tissue and cells (Lonard and Smith, 2002). It is unclear whether its effect on AQP2 in the kidney is mediated *via* agonistic or antagonistic effects, but we have observed expression of GPER as well as ERα and ERβ in the UUO kidneys. In addition, we have recently demonstrated expression of all three ER subtypes in the kidney collecting ducts (Cheema et al., 2015). It has previously been demonstrated that estrogen can activate CaMKII in an ERα-dependent manner in primary hippocampal neurons (O’Neill et al., 2008). This estrogen-induced CaMKII activity results in CREB phosphorylation without stimualtion of the cAMP, which is normally believed to be involved in CREB activation. Compatible with this, several studies have shown that CREB phosphorylation can stimulate AQP2 expression (O’Neill et al., 2008) and we have recently shown that TAM increases the pCREB/CREB ratio in IMCD tubule suspensions prepared from lithium-treated NDI rats, where AQP2 downregulation was attenuated (Tingskov et al., 2018). Taken together, we speculate that TAM-induced activation of CaMKII might induce AQP2 expression and trafficking *via* phosphorylation of CREB in a cAMP-independent manner. However, further studies are needed to elucidate this pathway in detail.

Interestingly, Zou and co-workers have previously identified a functional ER response element (ERE) in the AQP2 promotor in endometrial carcinoma cells (Zou et al., 2011). ERE can mediate the estrogen-mediated regulation of AQP2 in normal endometrium as well as in endometrial carcinoma cells (Zou et al., 2011). A similar molecular mechanism could underlie the regulation of AQP2 in the renal collecting ducts as well, and our unpublished data also showed that estradiol has a synergistic effect on dDAVP-induced AQP2 expression in mpkCCD cells. Precisely how the ERE in the AQP2 promoter contributes to the regulation of AQP2 transcription needs to be studied, but we cannot rule out the possibility that TAM can also affect AQP2 expression *via* the ERE. Coincidently, we have demonstrated increased expression of AQP2 in both cortical and medullary tissue from mice lacking ERα as compared to wild-type littermates (Cheema et al., 2015), indicating that the ERα receptor subtype might play an important role in the direct regulation of the *AQP2* gene. In view of that, we observe that the ERα receptor subtype is the most abundant ER in the UUO kidney and that TAM tends to reduce ERα mRNA expression and the short isoform of ERα. In addition, it has been demonstrated that TAM can act both as an agonist and antagonist for ERα (Barkhem et al., 1998), so it is also likely that TAM can affect the regulation of AQP2 *via* a combination of both agonistic or antagonistic effects, which might be dose-dependent. We have recently published that TAM does not increase AQP2 protein levels in IMCD cell suspensions with a TAM concentration at 1 and 5 nM (Tingskov et al., 2018), whereas the present data show that TAM at a concentration of 100 nM can induce AQP2 expression and trafficking in both normal and TGFβ-treated IMCD cells as well as in IMCD tubule suspension prepared from both sham-operated control and UUO rats.

## Conclusion

We have shown that TAM induces targeting of AQP2 to the apical plasma membrane and increased protein abundance of AQP2 under both normal conditions and pathophysiological circumstances, using a UUO model. In the primary cultured IMCD cells, TAM-induced membrane targeting and protein abundance of AQP2 was associated with phosphorylation at serine 256, the PKA-consensus phosphorylation site. However, TAM-stimulated increase of AQP2 abundance was not mediated through V2R and is largely independent of cAMP/PKA activation. On the other hand, TAM markedly increases CaMKII expression in the inner medullary renal tissues in both sham-operated control rats and UUO rats, which is likely to be an explanation for TAM-mediated regulation of AQP2. This study provides another example of AVP-independent regulation of renal AQP2.

## Data Availability

The datasets generated for this study are available on request to the corresponding author.

## Ethics Statement

The animal study was reviewed and approved by The Danish National Guidelines for care and the Danish veterinary and food administration (Approval no. 2015-15-0201-00658). The animal protocols were approved by the Animal Care and Use Committee of the Kyungpook National University, Korea (KNU 2012-10).

## Author Contributions

ST, H-JC, SH, CL, WW, JF, T-HK, and RN conceived and designed the research and interpreted the results of experiments. ST, H-JC, SH, and MH performed the experiments. ST, H-JC, SH, MH, LN, T-HK, and RN, analyzed the data. ST, H-JC, SH, and RN prepared the Figures. ST, H-JC, SH, T-HK, WW, and RN drafted the manuscript. ST, H-JC, MH, SH, CL, WW, JF, LN, T-HK, and RN edited and revised the manuscript and approved the final version of manuscript.

### Conflict of Interest Statement

The authors declare that the research was conducted in the absence of any commercial or financial relationships that could be construed as a potential conflict of interest.
